# Protein S for Portal Vein Thrombosis in Cirrhotic Patients Waiting for Liver Transplantation

**DOI:** 10.3390/jcm9041181

**Published:** 2020-04-20

**Authors:** Hao-Chien Hung, Jin-Chiao Lee, Chih-Hsien Cheng, Yu-Chao Wang, Tsung-Han Wu, Chen-Fang Lee, Ting-Jung Wu, Hong-Shiue Chou, Kun-Ming Chan, Wei-Chen Lee

**Affiliations:** 1 Division of Liver and Transplantation Surgery, Department of General Surgery, Chang-Gung Memorial Hospital, Chang-Gung University College of Medicine, Taoyuan 333, Taiwan; mp0616@cgmh.org.tw (H.-C.H.); b9302012@cgmh.org.tw (J.-C.L.); chengcchj@cgmh.org.tw (C.-H.C.); awuang726@gmail.com (Y.-C.W.); wutsunghan@gmail.com (T.-H.W.); lee5310@cgmh.org.tw (C.-F.L.); wutj5056@gmail.com (T.-J.W.); Chouhs@cgmh.org.tw (H.-S.C.); Chankunming@cgmh.org.tw (K.-M.C.); 2 Division of Liver and Transplantation Surgery, Department of General Surgery, Chang-Gung Memorial Hospital, 5, Fu-Hsing Street, Kwei-Shan, Taoyuan 333, Taiwan

**Keywords:** protein S, portal vein thrombus, cirrhosis, liver transplantation

## Abstract

Portal vein thrombus (PVT) is a challenge in liver transplantation. How PVT develops in cirrhotic patients who already have coagulopathy is unclear. This study aimed to investigate possible contributing factors to PVT in cirrhotic patients. A total of 349 cirrhotic patients who waited liver transplantation were included in this study and 48 of them had PVT. For all the patients, the mean age was 53.5 ± 9.0 year old, and 75.9% of the patients were male. There were 233 (66.8%) patients who had either hepatitis B or C. The mean Model For End-Stage Liver Disease (MELD) score was 16.4 ± 7.5. Eighteen of 48 patients with PVT and 145 of 301 patients without PVT received liver transplantation. Multivariate analysis showed that low protein S level (hazard ratio = 2.46, *p* = 0.017) was the only independent risk factor for PVT development. Protein S deficiency also demonstrated prognostic value on short-term survival, not only for cirrhotic patients awaiting liver transplantation (69.9% versus 84.1% at 1 year survival, *p* = 0.012), but also for the patients having liver transplantation (70.4% versus 84.8% at 1 year survival, *p* = 0.047). In conclusion, protein S level was an independent risk factor for PVT development in decompensated cirrhotic patients, and protein S deficiency was also a prognostic factor for the patients waiting for liver transplantation.

## 1. Introduction

Portal vein thrombosis (PVT) may lead to profound liver dysfunction, refractory ascites, gastrointestinal tract hemorrhage, and thrombotic ischemia in bowels [1,2]. If a patient has cirrhosis, PVT undoubtedly is a poor prognostic factor for this cirrhotic patient. The prevalence of PVT in cirrhotic patients varies from less than 1% in compensated cirrhosis [3] to around 15–20% in decompensated patients [4] depending on different underlying diseases and cirrhosis severities. General speaking, PVT presents as a late complication in cirrhosis progression [5], and creates a challenging clinical situation in liver transplantation. However, how PVT develops in a cirrhotic liver in a coagulopathy circumstance is not clear.

Liver dysfunction reduces both procoagulant and anticoagulant proteins under a complicated mechanism [6]. Generally, natural protein deficiency is rare and the prevalence rate is less than 1%, but the annual incidence of venous thromboembolism is up to 2% in cirrhotic patients [7]. With decompensated cirrhotic liver, patients often display attenuated coagulation functions, but may have deficiency in natural anticoagulant proteins. Thereafter, despite common bleeding tendency being noted in advanced cirrhotic patients, a hypercoagulable state may happen under an enhanced thrombin generation and prothrombotic situation [8]. All these reflect that PVT formation in cirrhosis is multifactorial and difficult to comprehend. Inherited and acquired factors, and hemodynamic hazards may all contribute to hypercoagulable state [9,10]. Previous studies postulated that male gender, history of splenectomy, portocaval shunting operations, ascites, presence of bleeding varices, thrombocytopenia, and advanced cirrhosis status (Child–Pugh class C) were all predisposing factors for PVT [5,11].

Reduction in circulating natural anticoagulant proteins was found in PVT patients [12], but it is unclear whether reduction of natural anticoagulant proteins is one of major factors in the development of PVT in cirrhotic livers. Because liver transplantation becomes difficult when high-grade PVT exists, it is better to identify the transplant candidates who may have the risk to develop PVT and it is also better to treat these high-risk patients prior to liver transplantation. However, the patients on waiting lists who have the tendency to develop PVT have rarely been paid much attention to before. In this study, we aim to understand possible factors that may predispose to PVT formation in cirrhotic patients and assess the outcomes after transplantations.

## 2. Materials and Methods

### 2.1. Patients

Between January 2013 and December 2015, 349 cirrhotic patients who waited for liver transplantation in our institute were reviewed retrospectively. The patients with hemostatic disorders such as portosystemic shunts, peripheral venous thrombosis history, use of oral contraceptives, anticoagulants or anti-platelet medication prior to investigation, or inherited coagulation abnormalities were excluded. PVT was defined by preoperative radiological studies and by presence of portal thrombosis intraoperatively if the patients had liver transplantation. The patients were categorized into two groups: PVT patients (*n* = 48) and non-PVT patients (*n* = 301). This study protocol conformed to the ethical guidelines of the 1975 Declaration of Helsinki and was approved by institutional review board of Chang-Gung Memorial Hospital (IRB No.20171264B0). Organs from executed prisoners were not used in this manuscript.

### 2.2. Clinical Examination and Data Collection

All liver transplantation candidates were assessed to fit the transplantation criteria, and the model for end-stage liver disease (MELD) scores were recorded. If the patients had hepatocellular carcinoma (HCC), HCC should be within the Milan criteria for deceased liver transplantation or the University of San Francisco (UCSF) criteria for living donor liver transplantation. Laboratory studies included blood cell count, platelet count, international normalized ratio (INR) of prothrombin time, protein C, protein S, albumin, creatinine, aspartate aminotransferase (AST), alanine aminotransferase (ALT), alkaline phosphatase (ALK-P), and serological tests for hepatitis B, hepatitis C, cytomegalovirus, and human immunodeficiency virus (HIV). Contrast-enhanced dynamic computed tomography (CT) was performed to assess portal vein patency, ascites status, grading of esophageal varices, and HCC status if presented. Presence and grading of esophageal varices were evaluated by endoscopy. The MELD scoring system was used to assess the severity of liver disease [13]. Portal flow was measured at three time points: pre-transplantation, intra-operation after portal vein (PV) reconstruction, and post-transplant day (POD) 1. The pre- and postoperative portal flow was measured using a duplex ultrasound, while intraoperative portal inflow was measured by electromagnetic flowmetry. The Clavien-Dindo classification was used for documenting post-transplant surgery complications [14]. A severe postoperative complication was defined as a grade greater or equal to III, and hospital mortality was defined as the patients who died during the same course of hospitalization for transplantation.

### 2.3. Statistical Analysis

Pearson’s chi-square test was used for categorical variables between the two groups (PVT versus non-PVT). Independent T test was used to compare clinical continuous parameters. The binary logistic regression model was used for univariate and multivariate analyses, and variables with *p* < 0.1 at univariate analysis were entered into further multivariate analyses to identify independent risk factors. Kaplan–Meier method was used to assess patient survival, and the differences between subgroups were analyzed by the log-rank test. A *p*-value <0.05 was considered as a significant difference. The SPSS (Version 24.0. Armonk, NY, USA: IBM Incorporation) software was used for all analyses.

## 3. Result

### 3.1. Patients

A total of 349 cirrhotic patients who were waiting liver transplantation and met the inclusion criteria are shown in Table 1. The mean age for the patients was 53.5 ± 9.0 years. Of the 349 patients, 265 (75.9%) patients were male. There were 233 (66.8%) patients who were infected with hepatitis: 43.2% with hepatitis B virus (HBV), 19.7% with hepatitis C virus (HCV), and 3.7% with both. There were 148 (42.8%) patients with HCC that met the Milan or UCSF criteria for deceased or living donor liver transplantation, respectively [15,16]. The mean MELD score of all patients was 16.4 ± 7.5. One-third of the patients experienced at least one episode of esophageal varices (EV) bleeding. Spontaneous porto-systemic shunt, either engorged coronary vein or splenorenal shunt, was found in 211 (60.5%) patients. Relatively low serum levels of protein C and protein S were observed in 262 (75.1%) and 48 (13.8%) patients, respectively. Low serum levels of protein C and protein S were correlated to disease entities, and the results showed that low levels of protein C and S were not associated with liver disease etiologies/HCC.

### 3.2. Clinical Difference between Patients with or without PVT

The incidence of PVT in cirrhotic patients was 13.75% in this study. There were no significant differences in age, gender, hepatitis status, HCC, ascites, and MELD score between PVT (*n* = 49) and non-PVT (*n* = 301) group patients. A total of 229 (65.6%) patients had EV in this study. The incidence of EV was higher in PVT group than in non-PVT group patients (79.2% versus 63.5%, *p* = 0.033), and the experience of EV bleeding was higher in PVT group than in non-PVT group (47.9% versus 25.9%, *p* = 0.001). Between PVT and non-PVT group, platelet count and serum levels of protein C and protein S were significantly different (Table 1).

### 3.3. Risk Factors of PVT Development

To identify the risk factors for development of PVT in cirrhotic patients when they were waiting for transplantation, the difference of clinical factors between PVT and non-PVT group was analyzed. Univariate analysis showed that platelet count ≤100 × 10^3^/uL (*p* = 0.051, hazard ratio (HR) = 1.66, 95% CI = 0.99–3.94), protein C deficiency (*p* = 0.017, HR = 3.22, 95% CI = 1.23–8.41), protein S deficiency (*p* = 0.005, HR = 2.82, 95% CI = 1.66–5.84), and presence of esophageal varices (*p* = 0.037, HR = 2.19, 95% CI = 1.05–4.56) were the significantly different factors between the two groups. In multivariate analysis, protein S deficiency was the only independent risk factor (*p* = 0.017, HR = 2.46, 95% CI = 1.17–5.46) (Table 2).

### 3.4. Diagnostic Accuracy of PVT on Imaging Study

Among 349 patients on waiting, 163 patients had either deceased or living donor liver transplantation. Among these patients, 13 patients had PVT and 150 patients did not have PVT, diagnosed by pre-transplant imaging study. During the operation, all 13 patients who had PVT diagnosed before transplantation had PVT. In the other hand, 5 among the 150 patients who did not have PVT prior to transplantation had PVT. Therefore, the sensitivity of preoperative radiological studies for PVT was 72.2%, and specificity was 100%. This indicated a perfect positive predict value for radiological studies, and the negative predict value for the exclusion of PVT was 96.7%.

### 3.5. Liver Transplantation

Of all enrolled patients, 163 patients, 145 non-PVT patients and 18 PVT patients, had liver transplantation, including 36 (22.1%) deceased donor liver transplant (DDLT) and 127 (77.9%) living donor liver transplantation (LDLT). The operation hours and intraoperative blood loss were not different between PVT and non-PVT patients (*p* = 0.899 and 0.459, respectively). Among 18 patients with PVT, 16 patients underwent thrombectomy, and portal vein was reconstructed as end-to-end manner. For the two patients failed to have thrombectomy, one patient had an interposition graft (cryopreserved iliac vein) from recipient coronary vein to graft portal vein, and the other patient had an interposition graft from recipient dilated middle colic vein to graft portal vein. In spite of limited cases of liver transplantation for the patients with PVT, the patients with PVT tended to have DDLT and ligate spontaneous shunts during operation simultaneously (*p* = 0.068 and 0.076, respectively). After operation, a higher proportion of PVT patients used anticoagulant agents to prevent thrombus reformation compared with the patients without PVT (33.3% versus 2.1%, *p* < 0.001; Table 3).

### 3.6. Measurement of Portal Flow

Among the 18 PTV patients with liver transplantation, 15 patients (72.2%) had grade 1–2 PVT and the other 3 patients had grade 3–4 PVT. Preoperative portal flow measured by Doppler ultrasound was 408.2 ± 155.8 mL/min. Portal flow was improved properly from 408.2 ± 155.8 mL/min to 1008.9 ± 218.0 mL/min in PVT patients after liver grafts were implanted. There was no difference of portal flow between PVT and non-PVT patients intra- and postoperatively (Table 3).

### 3.7. Outcomes after Liver Transplantation

Here were 77 (47.2%) patients who had various surgical complications (Table 3). The complication rates between PVT and non-PVT patients were not different. Hospital mortality occurred in 18 (11.0%) patients in this study, including 10 sepsis, 3 acute antibody-mediated rejection, 2 poor portal flow perfusion, and one small-for-size. Hospital mortality between PVT and non-PVT patients was not different. Regarding the patients with protein S deficiency, post-transplant complications happened in 63% of patients with protein S deficiency compared with 44.9% of non-protein S deficiency patients. Severe complications (≥grade III) were present in 40.7% of the patients with protein S deficiency and in 28.7% of the patients without protein S deficiency. By univariate analysis, low protein S level (*p* = 0.003, HR = 4.79, 95% CI = 1.73–13.41), high MELD score (*p* = 0.084, HR = 2.58, 95% CI = 0.88–7.53), intraoperative excessive blood loss (*p*= 0.001, HR= 5.85, 95% CI = 2.14–15.96), presence of EV (*p* = 0.082, HR = 3.11, 95% CI = 0.87–11.15), and low post-LT day 1 portal inflow (*p* = 0.077, HR = 2.45, 95% CI = 0.91–6.59) were the risk factors for hospital mortality (Table 3). Further multivariate analysis revealed that protein S deficiency (*p* = 0.009, HR = 4.58, 95% CI = 1.47–14.32) and excessive intraoperative blood loss (*p* = 0.09, HR = 4.33, 95% CI = 1.43–13.05) were the independent risk factors for hospital mortality (Table 3).

### 3.8. Survival

**Figure 1:**
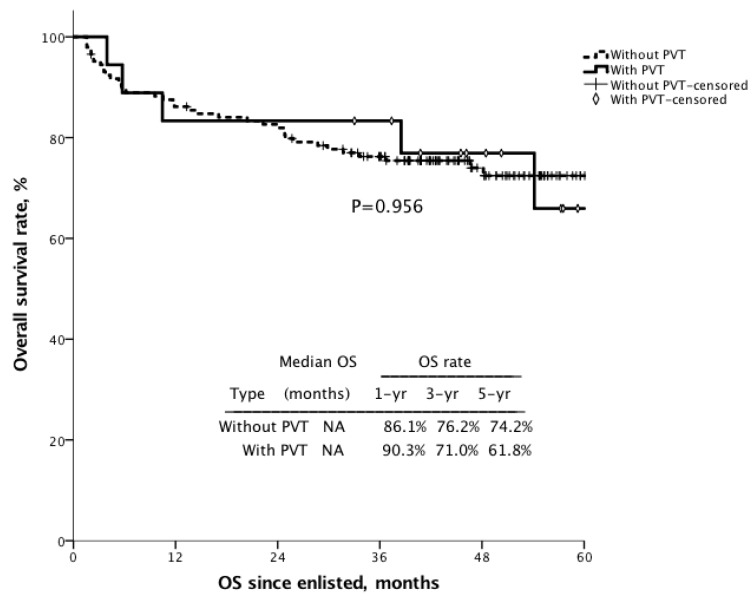
The Kaplan–Meier survival curve of the transplant recipients with or without PVT. The 1 year, 3 year and 5 year survival after transplantation for the patients with PVT were 90.3%, 71.0%, and 61.8%, compared with 86.1%, 76.2%, and 74.2% for the patients without PVT (*p* = 0.956).

**Figure 2:**
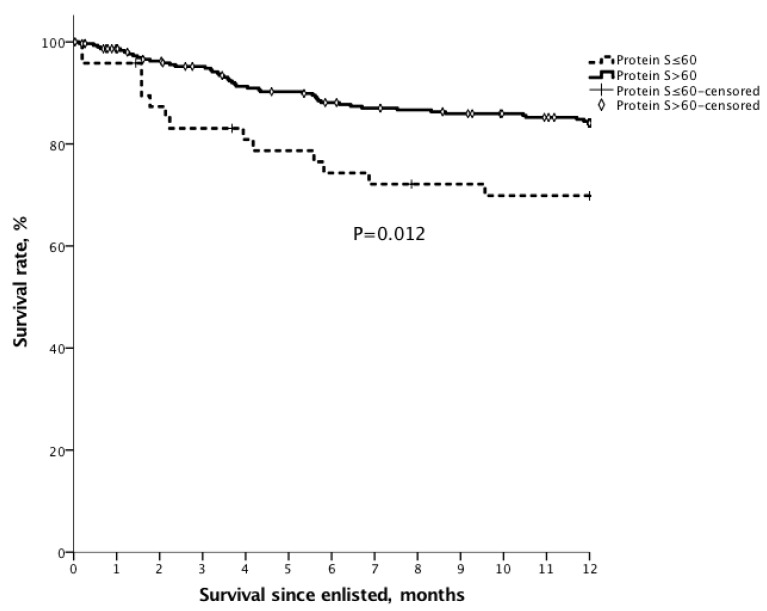
The Kaplan–Meier survival curve of patients waiting for liver transplantation with protein S ≤ 60%. For all enlisted patients, the 6 month and 1 year overall survival for the patients with protein S ≤ 60% (*n* = 48) were 74.3% and 69.9%, compared with 88.1% (*p* = 0.008) and 84.1% (*p* = 0.012) for the patients with protein S > 60%, respectively.

**Figure 3:**
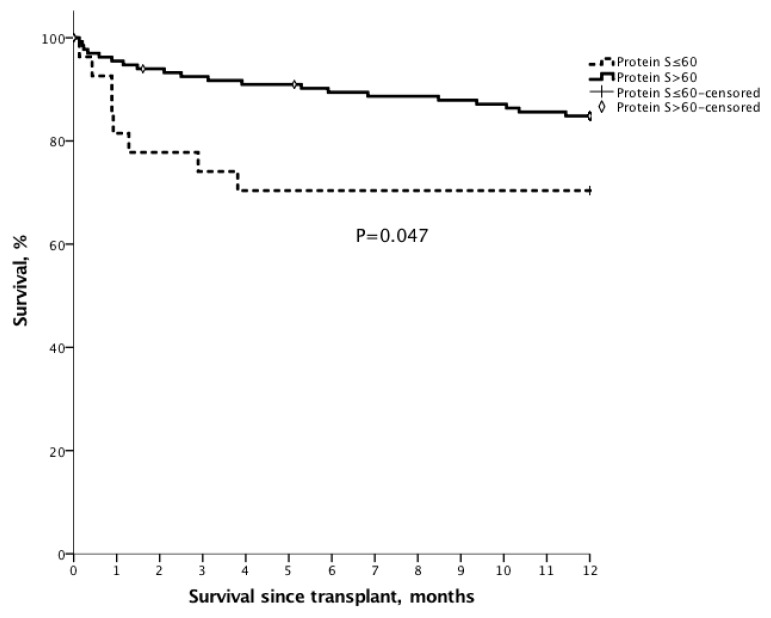
The Kaplan–Meier survival curve of patients with liver transplantation and protein S ≤ 60%. For the patients with transplantation, the 6 month and 1 year overall survival for the patients with protein S ≤ 60% (*n* = 27) were 70.4% and 70.4%, compared with 89.4% (*p* = 0.006) and 84.8% (*p* = 0.047) for the patients with protein S > 60% (*n* = 136).

The 1 year, 3 year and 5 year survival after transplantation for the patients with PVT were 90.3%, 71.0%, and 61.8%, compared with 86.1%, 76.2%, and 74.2% for the patients without PVT (Figure 1, *p* = 0.956).To assess the impact of protein S deficiency on survival, survival rates were analyzed for the patients with or without protein S deficiency in cirrhotic patients who were waiting for liver transplantation. For all enlisted patients, the 6 month and 1 year overall survival for the patients with protein S ≤ 60% (*n* = 48) were 74.3% and 69.9% compared with 88.1% (*p* = 0.008) and 84.1% (*p* = 0.012) for the patients with protein S > 60%, respectively (Figure 2). For the patients with transplantation, the 6 month and 1 year overall survival for the patients with protein S ≤ 60% (*n* = 27) were 70.4% and 70.4%, compared with 89.4% (*p* = 0.006) and 84.8% (*p* = 0.047) for the patients with protein S > 60% (*n* = 136) (Figure 3).

## 4. Discussion

Protein S deficiency significantly correlates with the development of PVT in this study. Reduction of natural proteins may indirectly explain the mechanism of PVT in decompensated cirrhosis. The pathogenesis of PVT in cirrhotic patients was not really clear until now. Decrement of portal flow after liver architectural destruction may be one of the reasons for PVT formation. When hepatitis attacks repeatedly, repeated inflammation can possibly trigger intrahepatic vein and sinusoids occlusion which results in congestive liver fibrosis, portal hypertension, and portal-systemic shunt [6]. The occurrence of PVT may develop precipitately either as a late consequence of cirrhosis or as a result of severely decompensated liver function [5,17]. Since synthesis of many coagulants and coagulation inhibitors takes place in the liver, it is important to balance the procoagulant and anticoagulant processes for physiological maintenance [18]. In cirrhotic livers with decompensated function, protein S deficiency may break the balance between procoagulant and anticoagulant processes, and cause PVT. In Dr. Chen’s previous study, the results did not show coagulation imbalance contributed to PVT formation in cirrhotic liver because comparisons of continuous variables between PVT and control group were utilized instead of categorized parameters [19]. Actually, low level of plasma natural proteins seems to be common in cirrhotic patients while waiting for liver transplantation. Although some studies [20,21] revealed a trend in protein S reduction in PVT group but failed to reach statistical significance, a recent meta-analysis [22] showed that protein S level was significantly lower in cirrhotic patients with PVT formation than in those without PVT formation. In addition, a combination of low plasma D-dimer and elevated protein S level is useful to exclude PVT possibility [23].

Protein S deficiency indicates the development of cirrhotic complications. The degree of liver function failure is related to development of impaired liver function tests, hyperbilirubinemia, hypoalbuminemia, prolongation of prothrombin time, thrombocytopenia, attenuated natural anticoagulant proteins, and ascites accumulation. In this study, prolongation of prothrombin time, thrombocytopenia, and relative deficiency of protein C and protein S levels were widely observed while these patients were waiting for liver transplantation. Clinically, protein S deficiency was associated with defective liver function and complications of liver dysfunction including high variceal degree, variceal bleeding, thrombocytopenia, prolongation of prothrombin time, hyperbilirubinemia, and high MELD score.

PVT is no longer recognized as a contraindication for liver transplantation [24]. The surgical techniques and caring strategies have been evolving coherently since the first successful attempt was performed by the Pittsburgh group in 1985 using a venous graft for reconstruction of PV flow [25]. However, post-transplant complications associated with PVT are still a major obstacle in liver transplantation, including re-thrombosis, re-operation, severe sepsis, acute kidney injury, hepatorenal syndrome, graft failure, and even death [5]. The occurrence of PVT has been reported in approximately 0.5–15% of patients with advanced cirrhosis who were waiting liver transplantation [26,27]. Our data revealed that the incidence of PVT was 13.8%, and 29.2% of them were with high-grade PVT. High-grade PVT may not only complicate with severe portal hypertension, massive ascites, variceal hemorrhage, and deterioration of liver function, but may also bring surgical challenges in PV flow establishment. In the literature, it was reported that overall complication occurred in more than half of the patients, with 46.2% infectious rate, 39% re-laparotomy, and 26.9% in-hospital mortality [27]. Our study revealed 2 of 18 (11.1%) patients with PVT were hospital mortalities. Therefore, PVT is no longer a contraindication for liver transplantation, but is still associated with high complication rates.

The survival of liver transplantation was comparable for the recipients with PVT or without PVT in this study. The survival was comparable for the recipients with PVT or without PVT in a meta-analysis study too [28]. Our surgical strategy for patients with grade I and grade II PVT in the Yerdel classification [5] was thrombectomy with PV end-to-end anastomosis. High-grade PVT may need dissection to low PV or even distal superior mesenteric vein (SMV), and then a jumping or interpositional vein graft is required between the graft and recipient. Our experience using a cryopreserved graft for portal reconstruction showed a satisfied result without immediate complication. Although survival was comparable for the transplant recipients with PVT or without PVT, the survival of the patients with PVT in waiting list was lower than that of the patients without PVT. Thus, the patients with protein S deficiency are associated with high PVT rate and favor to have liver transplant at an early time.

Many studies discussed the optimal PV flow after transplantation for adequate graft regeneration, but the result was inconclusive [29,30]. Different statements to this question were proposed between a wide range from 0.6–0.8 to 1.5–1.8 L/min. Under the situation of portal hypertension, portal-systemic shunting not only causes consumption and reduction of natural proteins, but also deteriorates already weakened portal flow to a pro-thrombus circumstance [31]. It could continue to influence the post-reconstructed portal flow. An insufficient reconstructed portal flow may fail a graft by regenerating setback, and a presence of hyperperfused portal inflow can lead to small-for-size syndrome (SFSS) with graft dysfunction, coagulopathy, and ascites [32]. Therefore, to maintain portal flow at an optimal perfusion is an early goal for post-transplant care.

A decline in the level of natural anticoagulant proteins secondary to cirrhosis with impaired liver function might cause PVT formation. In this study, protein S deficiency was more commonly found in the PVT group. Liver cirrhosis with portal hypertension results in endothelial damage and portal flow turbulences which further leads to a series of fibrolytic actions and subsequent consumption of natural proteins [33]. As an exclusive hepatocyte-synthesized substance, protein S acts as a cofactor for proteins C and a vitamin K-dependent thrombin-thrombomodulin complex that selectively inhibits activated-formed factors V and VIII [34]. Natural proteins deficiencies are found to be related with venous thromboembolism and put additional 3- to 10-fold risk in forming thrombosis [35]. In addition to advanced liver disease, acquired causes of protein S deficiency include any cause of disseminated intravascular coagulation, l-asparaginase, warfarin, cancerous condition, and acute severe bacterial infections, etc. In our practice, we do not routinely apply anticoagulant or anti-PLT in patients with PVT, but according to thrombus size, extension, degree of the occlusion, EV degree, bleeding history, recent episode, and risk of deterioration.

An idea about supplementation of anticoagulant proteins might be a resolution in cirrhotic patients with low levels of protein S [36]. It is still unclear whether anticoagulation does prevent re-thrombosis in high-risk patients after liver transplantation [27,37]. For protein C or S deficiency, cautiousness should be taken with warfarin as it also interferes with the production of natural anticoagulant proteins, and results in deterioration of the anticlotting ability for a short period. Rivaroxaban has been considered as another alternative treatment as a direct inhibitor of activated factor X without minifying anticoagulant ability of activated protein C [38]. Therefore, treatments for protein C or S deficiency are inconclusive.

The limitations of this study are that this study was conducted in a retrospective manner, and only small number of PVT patients had liver transplant. This is a cross-sectional study, and the results should be interpreted carefully. Further large-volume, longitudinal prospective studies are required in order to prove the validity of our findings. However, we believe that the result still can show the significant role of protein S in formation of PVT.

In conclusion, PVT is still a challenging condition for liver transplantation. This study showed that low protein S level was associated with PVT formation for cirrhotic patients waiting for liver transplantation. Although the outcomes of liver transplantation for PVT and non-PVT patients were not different, PVT increased the difficulty in portal flow reconstruction. Besides, PVT patients with low protein S (≤60%) have a worse survival while they are waiting for liver transplantation or after transplantation. Early liver transplantation for protein S deficiency patients may be beneficial.

## Figures and Tables

**Table 1:** Clinical characteristics of 349 cirrhotic patients with or without portal vein thrombus (PVT).

	Total, *n* = 349	Non-PVT, *n* = 301	PVT, *n* = 48	*p*-Value
Age, years	53.5 ± 9.0	53.7 ± 8.8	52.6 ± 10.4	0.429
Gender, male	265 (75.9%)	233 (77.4%)	32 (66.7%)	0.106
Hepatitis				0.718
HBV	151 (43.2%)	132 (43.8%)	19 (39.5%)	
HCV	69 (19.7%)	57 (18.9%)	12 (25.0%)	
Both	13 (3.7%)	12 (3.9%)	1 (2.0%)	
Alcohol use	130 (37.2%)	115 (38.2%)	15 (31.3%)	0.355
HCC, presence	148 (42.8%)	131 (44.0%)	17 (35.4%)	0.267
EV, presence	229 (65.6%)	191 (63.5%)	38 (79.2%)	0.033
EV bleeding history, presence	101 (28.9%)	78 (25.9%)	23 (47.9%)	0.001
Ascites, presence	192 (55.0%)	167 (55.5%)	25 (52.1%)	0.66
INR, >1.3	203 (58.2%)	172 (57.1%)	34 (64.6%)	0.332
PLT, ≤100 (10^3^/μL)	225 (64.5%)	187 (62.1%)	38 (79.2%)	0.022
Protein C, ≤70 (%)	262 (75.1%)	219 (72.8%)	43 (89.6%)	0.012
Protein S, ≤60 (%)	48 (13.8%)	35 (11.6%)	13 (27.1%)	0.004
Albumin, ≤2.8 (g/dL)	105 (30.1%)	94 (31.2%)	11 (22.9%)	0.244
Total bilirubin, >2 (mg/dL)	185 (53.0%)	159 (52.8%)	26 (54.2%)	0.863
ALK, >122 (U/L)	172 (49.3%)	151 (50.2%)	21 (43.9%)	0.408
Creatinine, >1.2 (mg/dL)	71 (20.3%)	62 (20.6%)	9 (18.8%)	0.768
e-GFR, ≤100 (mL/min/1.73m^2^)	169 (48.4%)	146 (48.5%)	23 (47.9%)	0.94
MELD score	16.4 ± 7.5	16.5 ± 7.8	15.9 ± 6.1	0.59
Spontaneous shunt	211 (60.5%)	181 (60.1%)	30 (62.5%)	0.755
Engorged CV	173 (49.6%)	146 (48.5%)	27 (56.3%)	0.319
Splenorenal shunt	107 (30.7%)	91 (30.2%)	16 (33.3%)	0.665
PVT grade, CT				NA
Grade 1–2			29 (60.4%)	
Grade 3–4			14 (29.2%)	

Abbreviation: HBV, hepatitis B virus; HCV, hepatitis C virus; HCC, hepatocellular carcinoma; INR, international normalized ratio; EV, esophageal varices; PLT, platelet; ALK, alkaline phosphatase; e-GFR, Glomerular filtration rate; MELD, model for end-stage liver disease; CV, coronary vein; PVT, portal vein thrombus; CT, computerized tomography. Quantitative values are expressed as mean ± SD. Categorial variables are expressed as frequencies, percentage.

**Table 2:** Univariate and multivariate analyses of cirrhotic patients by logistic regression on PVT.

	Univariate			Multivariate		
	HR	95%CI	*p*-Value	HR	95%CI	*p*-Value
PLT, PLT, 10^3^/μL						
≤100	1.66	0.99–3.94	0.051			
>100	1					
Protein C, %						
≤70	3.22	1.23–8.41	0.017			
>70	1					
Protein S, %						
≤60	2.82	1.36–5.84	0.005	2.46	1.17–5.16	0.017
>60	1			1		
Esophageal varices						
Absence	1					
Presence	2.19	1.05–4.56	0.037			

HR, hazard ratio; CI, confidence interval; PLT, platelet.

**Table 3:** Clinical characteristics of the patients with liver transplantation.

Liver Transplantation	Non-PVT, *n* = 145	PVT, *n* = 18	*p*
Op method			0.068
LDLT	116 (80.0%)	11 (61.1%)	
DDLT	29 (20.0%)	7 (38.9%)	
OP duration, hours	10.1 ± 2.0	10.2 ± 1.7	0.899
Blood loss, mL	2321 ± 2805	1825 ± 1100	0.459
Spontaneous shunt ligation	4 (2.8%)	2 (11.1%)	0.076
PV anastomosis			NA
PV–PV classic style	143 (98.6%)	16 (88.8%)	
CV–PV interposition style	2 (1.4%)	1 (5.6%)	
MCV–PV interposition style	0 (0.0%)	1 (5.6%)	
PVT grade, intraoperative			NA
Grade 1–2		13 (72.2%)	
Grade 3–4		5 (27.8%)	
PV flow assessment			
Pre-LT inflow, mL/min	561.4 ± 281.1	408.2 ± 155.8	0.095
Intraoperative inflow, mL/min	1517.3 ± 662.8	1360 ± 729.3	0.35
Post-LT day 1 inflow, mL/min	932.7 ± 258.8	1008.9 ± 218.0	0.233
Use of anticoagulant agent	3 (2.1%)	6 (33.3%)	<0.001
Postoperative complications			0.898
None	76 (52.4%)	10 (55.6%)	
Grade 1–2	26 (17.9%)	2 (11.1%)	
Grade 3–4	27 (18.6%)	4 (22.2%)	
Grade 5	16 (11.0%)	2 (11.1%)	
Acute rejection	11 (7.6%)	1 (5.6%)	0.756
Graft failure	8 (5.5%)	1 (5.6%)	0.995

Abbreviation: PVT, portal vein thrombus; LDLT, living donor liver transplantation; DDLT, deceased donor liver transplantation; OP, operation; PV, portal vein; CV, coronary vein; MCV, middle colic vein. Quantitative values are expressed as mean ± SD. Categorial variables are expressed as frequencies, percentage.

## Abbreviations

PVT	portal vein thrombus
MELD	model for end-stage liver disease
HCC	hepatocellular carcinoma
UCSF	University of San Francisco
INR	international normalized ratio
AST	Aspartate aminotransferase
ALT	alanine aminotransferase
CT	computed tomography
POD	postoperative day
HBV	hepatitis B virus
HCV	hepatitis C virus
EV	esophageal varices
DDLT	deceased donor liver transplantation
LDLT	living donor liver transplantation
